# Comparative proteomic analysis of horseweed (*Conyza canadensis*) biotypes identifies candidate proteins for glyphosate resistance

**DOI:** 10.1038/srep42565

**Published:** 2017-02-15

**Authors:** Fidel González-Torralva, Adrian P. Brown, Stephen Chivasa

**Affiliations:** 1 Department of Biosciences, Durham University, South Road, Durham, DH1 3LE, United Kingdom

## Abstract

Emergence of glyphosate-resistant horseweed (*Conyza canadensis*) biotypes is an example of how unrelenting use of a single mode of action herbicide in agricultural weed control drives genetic adaptation in targeted species. While in other weeds glyphosate resistance arose from target site mutation or target gene amplification, the resistance mechanism in horseweed uses neither of these, being instead linked to reduced herbicide uptake and/or translocation. The molecular components underpinning horseweed glyphosate-resistance remain unknown. Here, we used an *in vitro* leaf disc system for comparative analysis of proteins extracted from control and glyphosate-treated tissues of glyphosate-resistant and glyphosate-susceptible biotypes. Analysis of shikimic acid accumulation, *ABC-transporter* gene expression, and cell death were used to select a suitable glyphosate concentration and sampling time for enriching proteins pivotal to glyphosate resistance. Protein gel analysis and mass spectrometry identified mainly chloroplast proteins differentially expressed between the biotypes before and after glyphosate treatment. Chloroplasts are the organelles in which the shikimate pathway, which is targeted by glyphosate, is located. Calvin cycle enzymes and proteins of unknown function were among the proteins identified. Our study provides candidate proteins that could be pivotal in engendering resistance and implicates chloroplasts as the primary sites driving glyphosate-resistance in horseweed.

*Conyza* spp. (Asteraceae family) are highly invasive problematic annual weed species reported in more than 40 different crops in 70 countries^1^,^2^. These plants are very prolific, with estimations of a single horseweed plant producing up to 200,000 seeds, which are easily dispersed by wind to infest new territory^1^,^3^. Another characteristic contributing to success of these plants is the ability to suppress growth and development of other plant species in non-native regions^4^. In species such as hairy fleabane (*C. bonariensis*), the seeds remain dormant for approximately three years^5^, necessitating the need for continuous weed control in agricultural fields over consecutive seasons. A common method of controlling *Conyza* spp. is the use of herbicides, such as glyphosate (N-[phosphonomethyl]glycine).

Glyphosate is an inhibitor of the shikimate pathway, a metabolic pathway essential for production of aromatic amino acids (phenylalanine, tyrosine and tryptophan). The target enzyme for glyphosate is the chloroplastic enzyme 5-enolpyruvylshikimate-3-phosphate synthase (EPSPS, EC 2.5.1.19), which converts shikimate-3-phosphate and phosphoenolpyruvate to 5-enolpyruvyl-shikimate-3-phosphate^6^. Blockade of the shikimate pathway via inhibition of EPSPS causes accumulation of shikimic acid, which is followed by chlorosis and eventually death of treated plant tissues. Glyphosate is a non-selective systemic herbicide with low toxicity to mammals^7^. Heavy reliance on and excessive use of glyphosate for many decades exerted selection pressure on *Conyza* spp., which have now developed glyphosate-resistance. Glyphosate-resistant biotypes of three common species of *Conyza (C. bonariensis, C. canadensis* and *C. sumatrensis*) have been reported in many countries, such as Australia^8^, Brazil^9^,^10^, Canada^11^, China^12^, Czech Republic^13^, Greece^14^,^15^, Spain^16^,^17^,^18^, and USA^19^,^20^. The mechanism by which glyphosate resistance works is being intensely researched.

Glyphosate resistance in *Conyza* spp. include mutation of target enzyme and reduced translocation. Substitution of a conserved proline residue by threonine in the *EPSPS2* gene of *C. sumatrensis*, together with a reduction in absorption, have been reported as essential for glyphosate resistance^18^. Vacuolar sequestration, through active transport of glyphosate by *ABC* transporters^21^,^22^, has been described as the main resistance mechanism in several glyphosate-resistant weeds, including *Conyza* spp.^23^. This is supported by glyphosate-induced up-regulation of *ABC* transporters mainly *M10* and *M11* genes observed in glyphosate-resistant *C. canadensis*^24^. Herbicide metabolism has also been implicated in a Spanish *C. canadensis* biotype, where glyoxylate sarcosine and AMPA were detected in the resistant biotype^17^. Other researchers have reported increased *EPSPS* mRNA expression levels in glyphosate-resistant populations of *Conyza* spp.^25^.

One approach to investigate the mechanisms of herbicide resistance is application of large-scale transcriptomics, metabolomics and proteomics technologies. While transcriptomics^26^ and metabolomics^17^ have already been used, there have not been any reported attempts to use proteomics to investigate glyphosate resistance. Such an approach can potentially identify protein networks that have an important role in glyphosate resistance. In this paper, we used 2-dimensional gel-based and mass spectrometric analyses to identify proteins differentially expressed in response to glyphosate treatments in glyphosate-resistant and -susceptible biotypes of *C. canadensis*.

## Results and Discussion

Glyphosate was introduced to the commercial agrochemicals market in nineteen-seventy four^27^ as a broad-spectrum non-selective pre-planting burndown herbicide and later on gained use in targeted application in crops^28^. The introduction of glyphosate-resistant crops in 1996 entrenched its market dominance as the best-selling herbicide^27^ and global sales are projected to hit USD8.79 billion by the year twenty-nineteen^29^. The market success of glyphosate is under threat from the emergence of glyphosate-resistant weeds^27^,^30^ and research efforts to understand the molecular basis for resistance are intensifying. The mechanism of glyphosate resistance in horseweed is not fully understood and here we have used proteomic analysis as an investigative tool for a comparative study of the effects of glyphosate on resistant and susceptible horseweed biotypes.

### *In vitro* experimental system

We used a previously published *in vitro* experimental system of leaf discs^24^,^31^ to generate glyphosate-treated tissues for protein extraction. In this system, leaf discs floating on glyphosate solutions take up the herbicide, which activates a molecular response similar to glyphosate-sprayed whole plants. However, the advantage of the leaf disc system is its reproducibility due to uniform exposure to herbicide across experiments. We confirmed the responsiveness of glyphosate-resistant (GR) and glyphosate-susceptible (GS) horseweed plants *in vitro* by evaluating glyphosate-induced shikimic acid accumulation, activation of genes encoding ABC-transporter proteins, and phytotoxicity.

**Figure 1:**
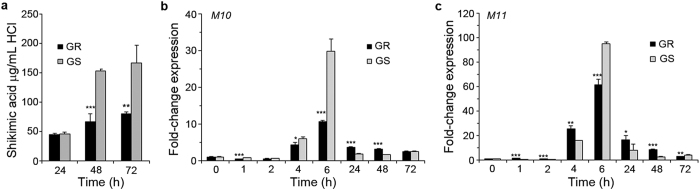
Activation of shikimic acid accumulation and *ABC-transporter* gene expression. (**a**) Time-course of shikimic acid accumulation in leaf discs of glyphosate-resistant (GR) and susceptible (GS) horseweed biotypes floated on 200 μM glyphosate. Bars represent mean ± s.d. (*n* = 5). (**b**) Quantitative-PCR analysis of *M10* gene (which encodes an ABC-transporter protein) in glyphosate-treated leaf discs harvested at the indicated time-points. (**c**) Quantitative-PCR analysis of *M11* gene (which encodes an ABC-transporter protein) in glyphosate-treated leaf discs harvested at the indicated time-points. Bars represent mean ± s.d. (*n* = 3). A significant difference between GR and GS is indicated by a single (*p* ≤ 0.05), two (*p* ≤ 0.01), or three asterisks (*p* ≤ 0.001).

In comparison to susceptible leaf tissues, there was a significant (*p* = 0.0086) suppression of shikimic acid accumulation in tissues from resistant horseweed plants exposed to glyphosate (Fig. 1a). Inhibition of EPSPS activity blockades metabolic flux through the shikimate pathway, resulting in the build-up of upstream intermediates, particularly shikimic acid^6^. Suppression of shikimic acid accumulation in glyphosate-resistant horseweed^24^ and *Conyza sumatrensis*^18^,^32^ biotypes has been reported previously. In fact, all glyphosate-resistant weeds examined to date somehow avoid accumulation of high levels of shikimic acid in comparison to susceptible plants, and this has been used as a diagnostic marker for resistance^33^.

Expression of genes encoding ABC-transporter proteins M10 and M11 had a bell-shaped profile, with a peak at 6 h in both GR and GS biotypes (Fig. 1b,c). ABC-transporter proteins can serve as xenobiotic efflux pumps^34^ and it has been suggested that higher activation of these genes in GR biotypes could account, at least in part, for horseweed glyphosate resistance^23^,^35^. However, our results show a stronger expression of these genes in GS than GR plants (Fig. 1b,c), suggesting that a role for these two proteins in glyphosate resistance of the biotype used in this study cannot be easily inferred from gene expression profiling data. Moreover, we noted that the peak expression of *M10* and *M11* preceded differential shikimic acid accumulation between the biotypes (Fig. 1). Furthermore, in contrast to our time-course analysis, previous studies have generally examined gene expression at one^22^,^24^ or two^36^,^37^ time-points. ABC-transporter gene expression was reported as higher in GR than GS at 24 h^22^,^24^,^36^,^37^, but to be the opposite at 96 h^36^,^37^. Although our detailed analysis shows a complex expression profile of *M10* and *M11* genes, it confirms that the *in vitro* system of floating leaf discs responds in a similar fashion to glyphosate-sprayed whole plants.

**Figure 2:**
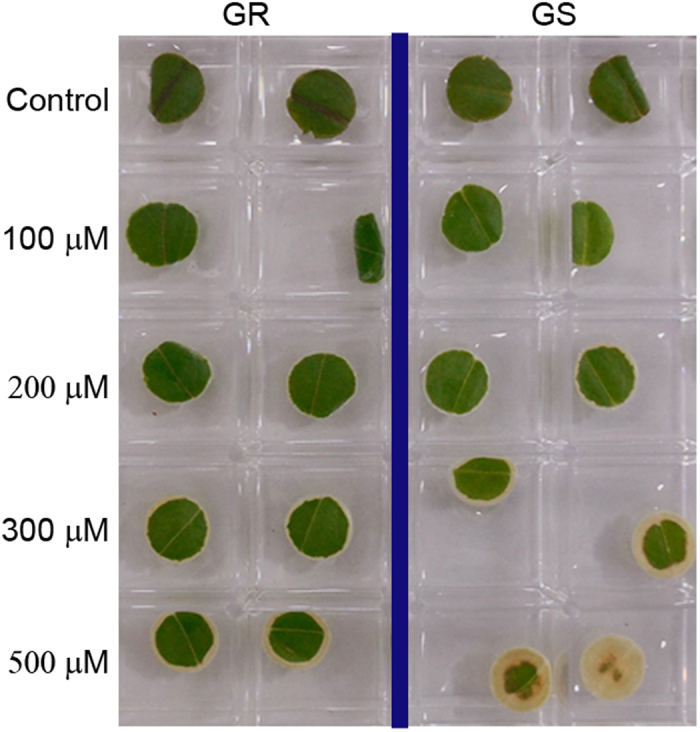
Glyphosate toxicity in horseweed biotypes. Leaf discs of glyphosate-resistant (GR) and susceptible (GS) horseweed biotypes were floated on glyphosate solutions with the indicated concentrations. The photograph was taken 72 h later.

Foliar spray application of glyphosate leads to cessation of growth and triggers chlorosis, which eventually culminates in plant death. At concentrations ≤200 μM glyphosate, leaf discs do not show significant symptoms within 72 h of treatment. Increasing the concentration triggers chlorosis and necrosis, which are far greater in the GS biotype than GR biotype as clearly seen in the 500 μM glyphosate treatment (Fig. 2). This reveals that glyphosate treatment of leaf disc tissue is attended by a biotype-specific response with regards to phytotoxicity. Overall, shikimic acid accumulation, *ABC-transporter* gene expression, and cell death profiles provide biochemical, molecular, and physiological evidence demonstrating the utility of the *in vitro* experimental system for use in studying glyphosate resistance mechanisms.

### 2D-DiGE analysis and protein identification

To identify molecular components underpinning glyphosate resistance in horseweed, we conducted comparative proteomics of GR and GS biotypes. We reasoned that the abundance of proteins with a putative function in resistance will either have different abundance between untreated tissues of the biotypes or exhibit differential response to glyphosate treatment. Protein samples were extracted from control and glyphosate-treated leaf discs 72 h after exposure to the herbicide. Each sample was an average of tissue from 5 independent plants to ensure representative results. We generated 4 independent biological replicates in order to account for biological variation and used a 2 dye labelling system, in which all samples are labelled with one dye (Cy3) and the pooled internal standard with the other dye (Cy5). This avoids technical variation arising from any potential discrepancies in labelling efficiency between the 2 dyes.

We made three main comparisons in quantitative analyses of protein spot abundance across the different sample groups. The first was control GS versus control GR, which reveals proteins with different abundance between the biotypes prior to glyphosate treatment. Second, was the control versus glyphosate-treated GS biotype. Lastly was the control versus glyphosate-treated GR biotype. From the latter two groups, we were particularly interested in identifying proteins whose response to glyphosate in the susceptible biotype was blocked in the resistant biotype and vice-versa. The image analysis software Progenesis SameSpots generated ratios from the normalised spot volumes and gave probability values associated with the analysis of variance and Student’s *t-tests*. Protein spots that were significantly (*p* ≤ 0.05) different between any pair-wise comparisons, for which we obtained positive identification, are presented with the related descriptive statistics in Supplementary Table S1. There is no complete genome sequence data for any species of *Conyza* in publicly available databases. By using sequence database searching with peptide precursor and fragment ion masses, proteins in families that are in common with related organisms can be easily identified, though gene identifications will await genome sequence data availability. Several studies have successfully used this approach on organisms with incomplete or completely non-existent full genome database^38^,^39^,^40^. Therefore, we searched all green plant sequences available in the TrEMBL database for related protein families. Proteins with the highest molecular weight search (MOWSE) score generated by the MASCOT search engine were selected. All protein identification data are provided in Supplementary Table S2.

### Comparison of untreated GR and GS biotypes

Very few proteins were identified in comparative analysis of GR and GS biotypes not exposed to glyphosate. Of the 17 differentially expressed protein spots, 3 were identified as having high homology to proteins of unknown function, with the remaining 14 belonging to the rubisco, ATP synthase, or fructose-bisphosphate aldolase protein families (Table 1). All these proteins are localised to the chloroplast, suggesting that the major differences between the GR and GS biotypes reside within this organelle. This observation is quite interesting, given that the shikimate pathway machinery, which is the target of glyphosate, is located in the chloroplasts. Perhaps follow-up proteomic experiments should analyse the chloroplast proteome using fractionation methods for chloroplast isolation. There was an overall down-regulation of chloroplast proteins in the GR biotype (Table 1). A total of 11 protein spots had decreased abundance in the GR biotype, with only 6 proteins having increased abundance. However, we noted that four spots (68, 73, 75, 77) with high homology to the large subunit of rubisco had a very low molecular weight than expected. It is quite curious that the higher molecular weight (~55 kDa) large subunit of rubisco spots (23, 24, 28, 30) were all down-regulated, while the lower molecular weight spots were all up-regulated (Table 1). Could this point to the possibility that the lower molecular weight spots are cleavage products of the higher molecular weight protein spots? Whether this result represents an authentic post-translational cleavage of large subunits of rubisco *in vivo* will await further analysis, but protein degradation during extraction is an unlikely explanation given that the tissues were homogenised in a solution with high levels of urea and thiourea. Moreover, the fact that this preferentially happens in the GR biotype would exclude protein extraction artefacts. A conjecture is that these low molecular weight products arise from the process of proteolytic turnover, suggesting that the GR biotype has a significantly different dynamic turnover of chloroplast proteins. Alteration of proteolysis rates is a recognised mechanism to diminish or augment protein pools^41^.

### Differential protein expression in response to glyphosate

Glyphosate at recommended field application rates is slow-acting, causing plant death 2–3 weeks after application, depending on the plant species. In our *in vitro* experiments, cell death activated by 200 μM glyphosate became apparent around 2 weeks post-treatment. We selected a sampling time-point of 72 h to capture the early shifts in protein abundance, occurring prior to appearance of cell death. Harvesting samples for protein extraction in dying cells could mask the physiologically important protein changes. Accordingly, the highest magnitude of protein abundance changes we observed at 72 h was ~2 fold (Table 2).

Between the 2 biotypes, we identified a total of 19 protein spots that responded to glyphosate treatment. They included photosynthetic enzymes, components of the translation machinery, a cytoskeletal protein, and proteins of unknown function (Table 2). We compared the response of these proteins to glyphosate between the 2 biotypes, searching for proteins whose response in one biotype was significantly different in the other biotype. Comparative analysis did not show any significant differences (*p* ≤ 0.05) in the glyphosate response of 16 of these proteins between the biotypes (Table 2). The observation that ~84% of the glyphosate-responsive proteins remained essentially unchanged between resistant and susceptible biotypes indicates that glyphosate resistance does not arise from a wholesale blockade of glyphosate-induced molecular events, but rather on inhibition of specific targets. However, there were 3 spots whose response to glyphosate in the susceptible biotype was significantly different in the resistant biotype, namely ATP synthase (spot 19), fructose-bisphosphate aldolase (spot 47) and an unknown protein (spot 51) (Table 2). The expression profile of these proteins in the horseweed biotypes is typical of what we expected of candidate proteins involved in glyphosate resistance.

### Potential role of proteins in glyphosate resistance

There is an overlap between proteins differentially expressed in response to glyphosate (ATP synthase, fructose-bisphosphate aldolase, unknown protein) and proteins with different abundance in untreated tissues of the biotypes (rubisco, ATP synthase, fructose-bisphosphate aldolase, unknown proteins). This is the expression profile one would expect from proteins with a function in mediating glyphosate resistance. With the exception of proteins of unknown function, the primary metabolic functions of the other enzymes are known and not associated with herbicide resistance. However, mounting evidence in the literature demonstrates that some enzymes of primary metabolism have secondary functions divorced from their known primary function and subcellular localisation. Therefore it is conceivable that some of these proteins could function in glyphosate resistance.

**Figure 3:**
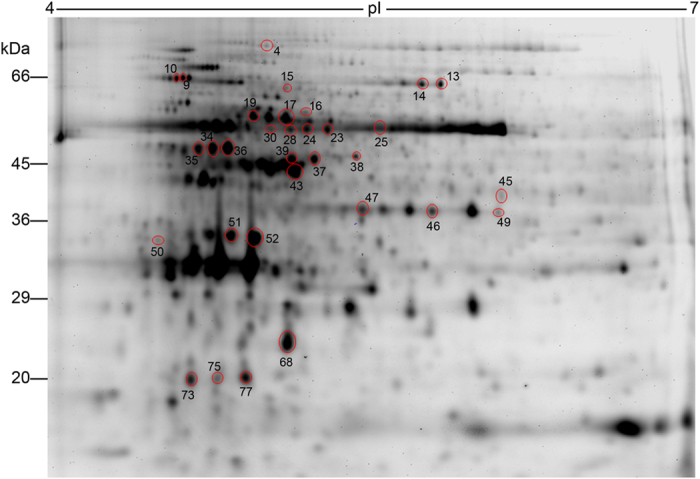
Two-dimensional gel image of horseweed proteins. Image shows 2-dimensional gel separation of a pool of proteins extracted from glyphosate-resistant and -susceptible horseweed biotypes and labelled with Cy5. The protein spots encircled in red were differentially expressed and the spot numbers correspond to numbers provided in Tables 1 and 2, which have data from the quantitative analysis.

Previous reports have demonstrated that reduced translocation could constitute a major aspect of glyphosate resistance in horseweed^35^. This could require the action of membrane transporter proteins for glyphosate transport to vacuoles or efflux into the apoplast. It has been suggested that tonoplast pumps in GR horseweed sequester glyphosate into the vacuoles, thus protecting the chloroplast and reducing the herbicide’s movement to young tissues^23^,^42^. How could proteins, such as the differentially expressed proteins we identified here, perform such a function? Fructose-bisphosphate aldolase catalyses the conversion of fructose-bisphosphate to glyceraldehyde-3-phosphate and dihydroxyacetone phosphate. The human equivalent ALDO A (mainly found in muscle cells) has an additional non-glycolytic function as a scaffold that links F-actin and the glucose transporter GLUT4 protein^43^. This scaffold connects GLUT4 vesicles to the cytoskeleton for trafficking to the plasma membrane after stimulation with insulin^43^. This enables the cell to heighten its uptake of glucose. A similar role for the plant aldolase we identified could influence glyphosate translocation via its influence on vesicle transport and exocytosis. Fructose-bisphosphate aldolase (spot 45) had reduced abundance in the glyphosate-resistant biotype (Table 1). In response to glyphosate treatment, the 2 aldolase spots (spots 46 & 47) significantly increased in the GS biotype, but only spot 46 (and not spot 47) increased in the GR biotype (Table 2). The two protein spots are charge variants of the same molecular weight (Fig. 3), raising the possibility of presence of a post-translational modification that could potentially regulate function. While, a function of aldolase in glyphosate transport is speculative at the moment, the precedence set in human cell glucose transport makes investigating a role in glyphosate resistance imperative.

ATP synthase subunits alpha, beta, and gamma make the F1 complex of the F0F1-ATP synthase motor protein responsible for cellular synthesis of ATP in chloroplasts and mitochondria. The subunits identified in this study are chloroplast proteins. We previously demonstrated that phytotoxicity triggered by the toxin fumonisin B1 in Arabidopsis requires ATP synthase β-subunit^44^. Mutant plants lacking a functional copy of this gene become resistant to the toxin and do not develop phytotoxic symptoms^44^. It is not yet clear if this resistance to toxin is related to translocation, detoxification, or efflux. However, ^31^P-Nuclear Magnetic Resonance studies support the idea that vacuole sequestration is ATP-dependent. Vacuole sequestration is lower when ATP levels are low, and under certain conditions the herbicide’s uptake is higher when ATP levels are expected to be at high levels^45^.

Though we have not been able to find any data in literature suggesting a secondary function of rubisco, we point to other proteins for which secondary functions have been identified. For example, cytochrome *c* is a mitochondrial electron-carrier essential for oxidative phosphorylation but, in response to cell death stimuli, it relocates to the cytosol where it is recruited to form an apoptosome together with Apaf-1 and caspase-9 to initiate apoptosis^46^. Moreover, glyceraldehyde-3-phosphate dehydrogenase (GAPDH) is a cytosolic enzyme involved in energy metabolism, but it also functions as a transcriptional regulator. It binds to and hitches a ride into the nucleus on E3-ubiquitin ligase, which possesses a nucleus localisation signal^47^,^48^. In the nucleus the GAPDH-E3-ubiquitin ligase complex binds to acetyltransferase p300/CREB binding complex, which enables a GAPDH-p300 subcomplex to directly bind promoters of the apoptosis proteins p21, p53, PUMA, and Bax^49^. Future studies will reveal if rubisco could similarly be hijacked in response to glyphosate or not.

## Conclusions

Horseweed is the first dicot weed species discovered to have evolved glyphosate resistance^19^, but its molecular resistance mechanisms remain unclear. It is now known that glyphosate translocates to root and shoot meristems, where it inhibits EPSPS and kills the plant^35^. Cellular uptake of glyphosate is suspected to be via active transporters when the concentration is low and via passive diffusion down a concentration gradient when applied at high concentrations^50^,^51^,^52^. Glyphosate translocation from sprayed leaves to meristems is impeded in GR horseweed biotypes^53^,^54^, most likely due to rapid vacuolar sequestration^23^,^42^. This might be a major component of a multi-layered resistance strategy with several biochemical and molecular processes. Our results have identified potential candidates that could be rapidly tested in model species, such as Arabidopsis, to investigate if transgenic plants overexpressing these genes have an altered response to glyphosate. These may or may not be involved in hindering glyphosate translocation/uptake, but the protein expression profile is consistent with a putative role in glyphosate resistance. We also observed a very clear enrichment of chloroplast proteins in the candidates differentially expressed in response to glyphosate or in GS and GR plant tissues not exposed to the herbicide. We conclude that this strongly suggests chloroplast proteins play a pivotal role in glyphosate resistance. Our future studies will focus on the chloroplast proteome by extracting protein fractions from chloroplasts isolated from the biotypes.

## Methods

### Plant material and growth conditions

Confirmed glyphosate-resistant (GR) and glyphosate-susceptible (GS) horseweed (*C. canadensis*) seeds were obtained from Delaware, USA. Seeds were directly sown in potted compost and the plants raised in a growth chamber with a 16 h photoperiod (100–120 μmol.m^−2^.s^−1^) maintained at 22/18 °C during the light/dark cycle, respectively. Plants were used for experiments when they reached the 8–12 leaf growth stage.

### Plant treatments and protein extraction

Leaf discs (8 mm-diameter) from GR or GS horseweed plants were floated on 8 mL of water (control) or 200 μM glyphosate (Sigma-Aldrich, Dorset, UK) in 5.5 cm-diameter Petri dishes. Four-replicate dishes for each of the four groups were generated: control and glyphosate-treated resistant biotype, plus control and glyphosate-treated susceptible biotype. This gave rise to a total of 16 dishes. All dishes had 10 leaf discs, each excised from an independent plant, to create a pooled sample with reduced biological variation. The Petri dishes were incubated under the same light and temperature regime as the plants from which they came. Leaf discs were harvested 72 h later, snap-frozen in liquid nitrogen, and stored at −80 °C until protein extraction. The leaf tissue was ground to a fine powder in liquid nitrogen and total soluble protein extracted in 700 μL of 9 M urea/2 M thiourea/4% CHAPS^55^. The protein was acetone-precipitated (80% acetone), pelleted by centrifugation (17,000 g, 10 mins), and washed 3 times with 80% acetone. The pellets were resuspended in labelling buffer (9 M urea/2 M thiourea/4% CHAPS/30 mM Tris-HCl pH 8.5).

### Quantitative protein analysis and identification

Differential expression analysis of proteins was performed using a 2-dye (Cy3 and Cy5) DiGE system as previously described^56^. Labelled protein samples were resolved in 24 cm-long pH 4–7 first dimension gels using the Ettan Ipgphor-3^TM^ system (GE Healthcare, Little Chalfont, UK) and separated in the second dimension using the Ettan DALT-12 system (GE Healthcare) following a protocol described before^57^. Gels were imaged with a Typhoon 9400 scanner (GE Healthcare) and image analysis performed using Progenesis SameSpots (Nonlinear Dynamics, Newcastle-upon-Tyne, UK). The software used normalised protein spot volumes to generate ratios for comparison of protein expression between selected pairs from the 4 sample groups (resistant control, resistant glyphosate-treated, susceptible control, susceptible glyphosate-treated). We were interested in 3 comparisons: control versus glyphosate-treated samples in both the susceptible and resistant biotypes, and susceptible control versus resistant control samples. Protein spots with a statistically (Student’s *t-test*) significant (*p* ≤ 0.05) difference in abundance between any of the 3 comparisons were selected for mass spectrometric analysis.

Protein spots of interest were robotically (Investigator^TM^ ProPic Robot, Genomic Solutions, Huntingdon, UK) excised from preparative 2D gels loaded with 800 μg total proteins and stained with Sypro® ruby (Genomic Solutions). The Investigator^TM^ Progest workstation (Genomic Solutions) was used for automated trypsin digestion of proteins inside the gel plugs using the overnight digestion protocol settings. In the first instance, all protein spots were analysed by tandem mass spectrometry using the 4800 *Plus* MALDI TOF/TOF Analyser (Applied Biosystems, Foster City, USA). Analysis was controlled by the instrument’s 4000 series Explorer software (version 3.5) set on automated data acquisition mode for TOF-MS analysis. The settings were; reflector mode, mass range 700–4000 *m/z*, 1000 total laser shots per spectrum, and laser intensity of 3300 V. Noise-correction and peak deisotoping were applied to the spectra, which were then internally calibrated using trypsin autolysis peaks 842.500 ad 2211.100 *m/z*. For MS-MS analyses, up to 10 of the most abundant precursor ions were automatically selected for 1 kV CID fragmentation, with 4000 laser shots per spectrum at an intensity of 3800 over the mass range being collected. GPS Explorer version 3.6 was used to generate peak lists of ion masses from the MS and MS-MS spectra post-calibration and de-isotoping.

Protein spots for which no identification could be established via the 4800 Proteomic Analyser were reanalysed by a hybrid quadrupole-TOF mass spectrometer (QStar Pulsar *i*, Applied Biosystems) coupled to a nanospray source (Protana) and a PicoTip silica emitter (New Objective, Woburn, MA) as described previously^58^. Sample digests loaded on a Zorbax 300SB-C18, 5 μm, 5 × 0.3 mm trap column (Agilent, Stockport, UK) were washed and online chromatographic separation on an Acclaim PepMap 100 C18 3 μm capillary column (25 cm × 75 μm) (Thermo Scientific, Cramlington, UK) performed with a 0–40% acetonitrile, 0.1% formic acid linear gradient over 2 h at a flow rate of 300 nL/minute. Analyst software version 1.1 (Applied Biosystems) performed all MS and MS/MS data acquisition, switching every 10 s between the survey scan (1 × 1 s MS) and three product ion scans (3 × 3 s MS/MS). Ions in the 2+ to 4+ charge state range and TIC > 10 counts were selected for fragmentation.

MS and MS-MS datasets were used to interrogate the TrEMBL plants-only database (2888720 sequences; 1026923278 residues) downloaded 15 October 2013. MASCOT search engine (version 2.1 for the 4800 Proteomic Analyser or version 2.5.1 for the QStar Pulsar *i*) from Matrix Science (MA, USA) was used with the following search parameters; protease enzyme trypsin, allowance for a single missed cleavage site, variable modifications of oxidised methionine and carbamidomethyl cysteine, 50 ppm precursor ion mass tolerance, and 0.2 Da fragment ion tolerance. Positively identified proteins had a protein score, incorporating MS-MS-derived individual ion scores and peptide mass fingerprint-associated score, higher than 95% (*p* ≤ 0.05). A decoy database (reversed sequences of the target database) search was included and False Discovery Rate was set at 1%.

### Shikimic acid assays

Five leaf discs from 5-replicate GR or GS plants were treated with 200 μM glyphosate or water (serving as controls) for up to 72 h. After harvesting, the discs were snap-frozen in liquid nitrogen in a microfuge tube containing 125 μL 0.25 N HCl and stored at −20 °C until further analysis. Shikimic acid determination was performed according to a previously described method^33^,^59^, with minor modifications. After thawing, samples were vortexed for 5 min and centrifuged (10,000 g, 5 min) and incubated at 60 °C for 15 min. Aliquots of 25 μL were transferred to new tubes and mixed with 100 μL of 0.25% (w/v) periodic acid/0.25% (w/v) m-periodate. Samples were then incubated at 37 °C for 30 min, after which 100 μL of 0.6 N sodium hydroxide/0.22 M sodium sulphite were added. Shikimic acid in the samples was determined spectrophotometrically at 382 nm against pure shikimic acid (Alfa Aesar, Heysham, UK) standards similarly processed. Glyphosate-induced shikimic acid accumulation was calculated by subtracting the basal levels in control leaf tissues from that of glyphosate-treated leaf discs. The experiments had four replicates, with each replicate consisting of 5 leaf discs from five different plants. Shikimic acid accumulation data were analysed using the Student’s *t*-test.

### RNA analyses

Leaf discs from GR or GS plants were treated with 200 μM glyphosate and harvested 0, 1, 2, 4, 6, 24, 48, or 72 h after treatment. Triplicate biological samples, each consisting of 5 leaf discs from 5 independent plants, were harvested at each time-point and used for RNA extraction. Total RNA was isolated using the RNA Spectrum™ Plant Total RNA Kit (Sigma-Aldrich), following the manufacturer’s instructions. First-strand cDNA synthesis via reverse transcription used 1 μg RNA, oligo (dT)_15_ (Promega, Southampton, UK) and SuperScript III reverse transcriptase (Invitrogen, Paisley, UK).

Real-time quantitative-PCR was performed using SensiFAST SYBR (2x)^®^ on a Rotor-Gene 3000 (Corbett Research, Sydney, Australia). The reaction was carried out with 1 μL of a 2-fold dilution of cDNA, 10 μL SensiFAST SYBR (2x)^®^ and 0.4 μM each of forward and reverse primers in a 20 μL reaction volume. Cycling conditions were as follows: hold temperature at 95 °C for 3 min, followed by 40 cycles: denaturation at 95 °C for 10 s; 56 °C for 15 s and an extension of 72 °C for 25 s. Primers were designed using the Primer3Plus software^60^ to amplify 99–110 bp of the target genes. The following primer pairs were used: *M10* 5′-GCAGGCCGTTAAGTGACAAT-3′ and 5′-TTGTGTTTCCACCGTTTTCA-3′; *M11* 5′-AAATTTGGGAGGCTCTCGAT-3′ and 5′-CGCTCCAGTTTTCTCCATTC-3′; *ACTIN* 5′-CCGATCCAGACGCTGTATTT-3′ and 5′-TGCTGATCGTATGAGCAAGG-3′. The *ACTIN* gene, whose expression does not change in response to glyphosate^22^, was used as a constitutive reference control gene. Primers for gene sequences of the ABC transporters *M10* and *M11* and *ACTIN* have been used previously^22^. Relative gene expression values were calculated using the Relative Expression Software Tool (REST v.2009)^61^ software by analysing the take off and amplification efficiency of each target gene relative to the *ACTIN* reference gene.

## Additional Information

**How to cite this article:** González-Torralva, F. *et al*. Comparative proteomic analysis of horseweed (*Conyza canadensis*) biotypes identifies candidate proteins for glyphosate resistance. *Sci. Rep.* **7**, 42565; doi: 10.1038/srep42565 (2017).

**Publisher's note:** Springer Nature remains neutral with regard to jurisdictional claims in published maps and institutional affiliations.

## Supplementary Material

Supplementary Information

## Figures and Tables

**Table 1:** List of proteins differentially expressed in control samples.

Spot No.^a^	TreMBL Accession^b^	Protein name	GR/GS	
			Ratio^c^	*p*-value^d^
4	R0F3T9	Uncharacterized protein	−1.5	0.029
15	Q0DA88	Os06g0669400 protein	−1.5	8.2e-5
16	Q1KXW5	ATP synthase subunit alpha, chloroplastic	−1.7	0.002
17	Q1KXW5	ATP synthase subunit alpha, chloroplastic	−1.5	0.025
19	Q1KXW5	ATP synthase subunit alpha, chloroplastic	−1.6	0.018
23	G0WXR8	Ribulose-1,5-bisphosphate carboxylase/oxygenase large subunit	−1.4	0.016
24	G0WZH2	Ribulose-1,5-bisphosphate carboxylase/oxygenase large subunit	−1.6	0.011
28	G0WXR8	Ribulose-1,5-bisphosphate carboxylase/oxygenase large subunit	−1.5	0.043
30	E3TI13	Ribulose-1,5-bisphosphate carboxylase/oxygenase large subunit	−1.4	0.029
45	J3N6C9	Fructose-bisphosphate aldolase	−1.3	0.018
49	M1PY75	Ribulose bisphosphate carboxylase large chain	1.3	0.026
50	G0WXR8	Ribulose bisphosphate carboxylase large chain	−1.4	0.044
51	M1AMG7	Uncharacterized protein	1.5	0.001
68	F8RP80	Ribulose-1,5-bisphosphate carboxylase/oxygenase large subunit	1.9	0.027
73	J9QFM2	Ribulose-1,5-bisphosphate carboxylase/oxygenase large subunit	1.9	0.023
75	J9QFM2	Ribulose-1,5-bisphosphate carboxylase/oxygenase large subunit	1.9	0.039
77	K9L5M8	Ribulose-1,5-bisphosphate carboxylase/oxygenase large subunit	1.9	0.032

^a^Spot number assigned during gel processing.

^b^Protein accession number in the TreMBL database.

^c^Ratio of the comparison of normalised spot volume in glyphosate-resistant (GR) to glyphosate-susceptible (GS) horseweed biotypes. Negative values indicate protein down-regulation in GR biotype.

^d^Probability value from Student’s *t-test* analysis.

**Table 2:** List of differentially expressed proteins during the response to glyphosate.

Spot No.^a^	TreMBL Accession^b^	Protein name	GS		GR		GR/GS^e^	
			Ratio^c^	*p*-value^d^	Ratio^c^	*p*-value^d^	Ratio^c^	*p*-value^d^
9	L0HT24	Heatshock protein 70	1.4	0.006	1.5	0.002	−1.1	0.308
10	L0HT24	Heatshock protein 70	1.4	3.9e-4	1.6	0.004	1.0	0.505
13	G7IF28	Transketolase	1.3	0.078	1.4	0.033	1.0	0.658
14	M5X661	Uncharacterized protein	1.3	0.027	1.5	0.002	1.2	0.090
19	Q1KXW5	ATP synthase subunit alpha, chloroplastic	1.0	0.928	1.3	0.006	−1.3	0.031
25	D3PFB6	Ribulose-1,5-bisphosphate carboxylase/oxygenase large subunit	−1.4	0.027	−1.2	0.102	1.1	0.129
34	K7RYG4	Chloroplast ribulose bisphosphate carboxylase/oxygenase activase beta2	1.4	5.6e-4	1.2	0.062	1.0	0.448
35	K7RYG4	Chloroplast ribulose bisphosphate carboxylase/oxygenase activase beta2	1.3	0.007	1.2	0.200	1.1	0.499
36	K7RYG4	Chloroplast ribulose bisphosphate carboxylase/oxygenase activase beta2	1.4	0.003	1.3	0.037	1.3	0.060
37	G5ELM7	Actin	1.2	0.025	1.3	0.018	1.1	0.129
38	M5XAL4	Elongation factor Tu	1.1	0.026	1.1	0.130	1.0	0.971
39	Q9XQ94	Glutamine synthetase leaf isozyme, chloroplastic	1.2	0.033	1.2	0.047	1.1	0.130
43	Q1KXW5	ATP synthase subunit alpha, chloroplastic	1.2	0.016	1.2	0.080	1.0	0.586
45	J3N6C9	Fructose-bisphosphate aldolase	1.1	0.279	1.3	0.046	1.1	0.184
46	M5VP21	Fructose-bisphosphate aldolase	1.3	0.004	1.4	0.006	1	0.439
47	B6TI65	Fructose-bisphosphate aldolase	1.2	0.029	1.1	0.420	1.3	0.044
49	M1PY75	Ribulose bisphosphate carboxylase, large chain	1.5	0.001	1.3	0.016	1.1	0.325
51	M1AMG7	Uncharacterized protein	−2.0	1.8e-5	−2.3	1.7e-4	1.3	0.029
52	I1NND9	Uncharacterized protein	−1.4	0.004	−1.8	0.005	1.0	0.954

^a^Spot number assigned during gel processing.

^b^Protein accession number in the TreMBL database.

^c^Ratio represents the average fold-change (*n* = 4) in abundance when comparing glyphosate-treated samples to controls in either the glyphosate-susceptible (GS) or glyphosate-resistant (GR) biotype. Negative values indicate protein down-regulation.

^d^Probability value from Student’s *t-test* analysis.

^e^Comparison of average spot abundance of glyphosate-treated GR and GS samples.
